# Bright Dots and Smart Optical Microscopy to Probe Intracellular Events in Single Cells

**DOI:** 10.3389/fbioe.2018.00204

**Published:** 2019-01-04

**Authors:** Hideaki Fujita, Chongxia Zhong, Satoshi Arai, Madoka Suzuki

**Affiliations:** ^1^WASEDA Bioscience Research Institute in Singapore, Singapore, Singapore; ^2^Institute for Protein Research, Osaka University, Osaka, Japan; ^3^Research Institute for Science and Engineering, Waseda University, Tokyo, Japan; ^4^PRIME-AMED, Tokyo, Japan; ^5^PRESTO, Japan Science and Technology Agency, Saitama, Japan

**Keywords:** fluorescent protein, nanoparticle, infrared, nanodiamond, quantum dot, Raman, temperature

## Abstract

Probing intracellular events is a key step in developing new biomedical methodologies. Optical microscopy has been one of the best options to observe biological samples at single cell and sub-cellular resolutions. Morphological changes are readily detectable in brightfield images. When stained with fluorescent molecules, distributions of intracellular organelles, and biological molecules are made visible using fluorescence microscopes. In addition to these morphological views of cells, optical microscopy can reveal the chemical and physical status of defined intracellular spaces. This review begins with a brief overview of genetically encoded fluorescent probes and small fluorescent chemical dyes. Although these are the most common approaches, probing is also made possible by using tiny materials that are incorporated into cells. When these tiny materials emit enough photons, it is possible to draw conclusions about the environment in which the tiny material resides. Recent advances in these tiny but sufficiently bright fluorescent materials are nextly reviewed to show their applications in tracking target molecules and in temperature imaging of intracellular spots. The last section of this review addresses purely optical methods for reading intracellular status without staining with probes. These non-labeling methods are especially essential when biospecimens are thereafter required for *in vivo* uses, such as in regenerative medicine.

## Introduction

**Figure 1:**
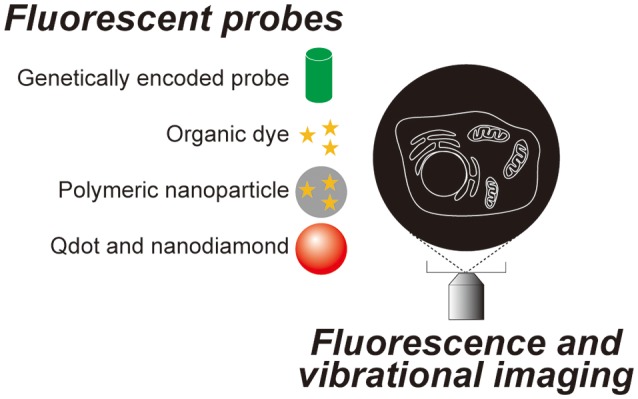
Fluorescent probes and optical microscopy techniques introduced in this review.

**Figure 2:**
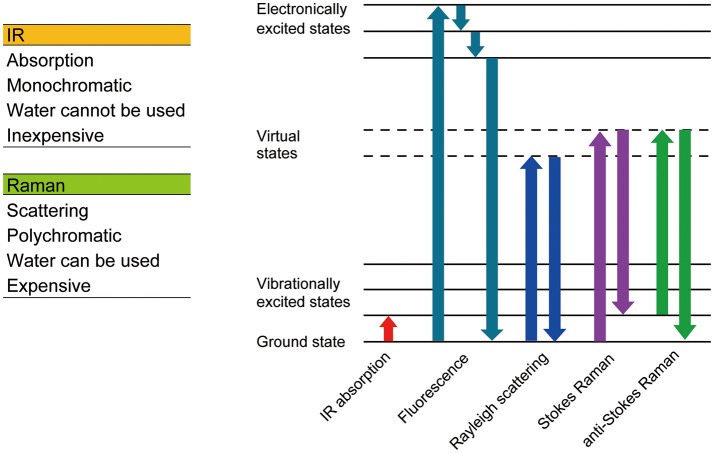
Comparison between Raman and IR spectrometry.

Since Robert Hooke observed a cork and named a compartment he found within it a “cell” using a simple microscope composed of two lenses in the 17th century, microscopic observation has become one of the most essential techniques in cell biology. Most animal cells are almost transparent and various techniques were developed to image these transparent cells. The simplest method is to label the cells with colored probes. Haematoxylin-Eosin staining was introduced back in the 19th century and it is still commonly used in histological labs for diagnostics. Now, there are many probes with various functions and characteristics (Figure 1), which are briefly summarized in section Genetically encoded probes vs. chemical dyes to highlight the variety. These fluorescent probes were not designed to work on single molecules. The signal is captured as an average value of many molecules. When many molecules are concentrated in a small space, a bright fluorescent nanoprobe can be obtained. When optical microscopy is optimized for nanoprobes, location tracking as well as environment probing are made possible using a single particle. Section Bright nano-dots as single probes for tracking of this review focuses on nanoprobes that are designed to be imaged one by one with a purpose to monitor intracellular parameters such as the location of biomolecules and spatial and temporal changes in temperature. Other methods do not involve staining. Differential interference contrast microscopy and phase contrast microscopy both utilize the phase shift between the illuminating light and the light which passes through the specimen and in so doing can visualize transparent cells without labeling. These are not discussed in this review as there is much less information on the cellular states that can be determined using these techniques when compared to the other methods we will discuss. Imaging utilizing the vibrational information of a molecule is discussed in section Imaging technologies without labeling of this review (Figure 2).

## Genetically Encoded Probes vs. Chemical Dyes

Fluorescent sensors are defined as those in which the signal, such as fluorescence intensity, wavelength, or lifetime, is altered in response to a change in the environment. They are called fluorescent probes, indicators, and sensors (hereafter referred to as “fluorescent probes”) (Zhang et al., 2002). The probes can be broadly categorized into two groups: genetically encoded probes and small chemical dyes. Which probe to choose depends on what intracellular events need monitoring and the duration of the observation.

### Genetically Encoded Fluorescent Probes

From the standpoint of long-term observation in fluorescence microscopy, genetically encoded probes are preferred. If we are keen to make observations for a few hours or days using small molecule indicators, we need to employ the strategy where the dyes are anchored to the target places or proteins covalently, which is still challenging (Wakayama et al., 2017). Also, to specifically observe an evenet of interest, genetically encoded probes once again prevail over small chemical dyes due to the working mechanism that they employ. The ability of natural proteins to bind to a specific target is adaptable in genetically encoded probes, while small chemical indicators use synthetic modules for sensing which are usually less specific to the target molecule than natural counterparts. Thus, genetically encoded probes exhibit better specificity to a target, especially against small molecules such as Ca^2+^, cAMP, ATP, and glucose (DiPilato et al., 2004; Imamura et al., 2009; Zhao et al., 2011; Hofig et al., 2018).

Regarding the delivery of probes into cells, genetically encoded probes require transfection using reagents, or physical delivery. To evaluate the probes for more than several days, preparation of a stable cell line into which the genetic code of the probe is integrated into the genome is necessary. Also, if transfection is difficult, such as in the case of primary cultured cells, viral infection is unavoidable. Another feature worth highlighting is that the location of the probes can be controlled at an organelle level. For example, a target sequence, such as for the nucleus (nuclear localization signal), mitochondria (subunit VIII of cytochrome c oxidase), or cellular membrane (growth-associated protein 43, neuromodulin), is conjugated to the N or C terminus of the probe, which then selectively localizes the probe to the target organelle (De Giorgi et al., 1999; Arai et al., 2018). Lastly, once the plasmid for a probe is purchased or given as a gift from the developer, it is always possible to amplify the plasmid at little cost.

### Organic Dyes

One may feel that genetically encoded probes are the perfect choice. However, there is one critical issue–ease of handling. Genetically encoded probes require around 1–3 days for expression in cells after delivery of the gene. On the other hand, small chemical indicators are easy to apply for staining, usually taking around 30 min to take effect before imaging can begin (Zhu et al., 2016). For those who rarely perform molecular biology protocols, transfection might be difficult to apply to their biospecimens or it may be that the use of viruses for infection is not allowed in their laboratory. In some cases, biological samples cannot withstand the 1 day incubation time needed for gene expression. In these instances small chemical dyes are the only remaining option. Across the field of research, imaging is often required for a variety of cell and tissue types, as well as different animal species, beyond that of commonly used cell lines (Vendrell et al., 2012). Gene delivery frequently proves to be a challenge under these conditions. Small chemical dyes can therefore prove to be helpful and even be considered the first choice for imaging. One can find in the literature positive examples of labeling using small near-infrared dyes in biomedical research at tissue and animal levels (Hong et al., 2017). The purpose of the experiment and the type of specimen should always be the primary factors in determining which probes to use, regardless of whether they are small chemical dyes, or genetically encoded probes.

These fluorescent probes were designed to take an average of many molecules to measure a given parameter. Thus, spatial resolution is limited by optical microscopy. Are there means to improve accuracy in location while probing parameters using optical microscopy?

## Bright Nano-dots as Single Probes for Tracking

The accumulation of fluorescent molecules creates a fluorescent nanoparticle. Quantum dots (Qdots) and nanodiamonds are also nanoparticles that emit photons upon excitation by light. These nanoparticles can be so bright that each dot is tracked in single camera frames repeatedly at 30 Hz or faster. When the nanoparticle is designed as a sensor, it is possible to probe the position of the nanoparticle as well as garner information about its local environment. If said probing is repeated frame by frame, time courses of the measured parameters can be obtained instantly. Among the large variety of applications of nanoparticles for probing, temperature sensing is one of the most advanced for biological usage. As the temperature is a fundamental parameter that can influence chemical and physical processes in any biological systems, the mechanism how living organism senses the temperature and how it releases heat in its body has been one of the major research topics in biology, and in animal, and plant physiologies. Recent advances in optical probes has brought our interest from the macroscopic (such as the temperature of the animal body or of the cell culture medium) to microscopic scale as small as a single cell. However, local event of heat release is still unclarified in live cells. Excellent reviews on fluorescent thermometers in general can be found in the literature (Brites et al., 2012; Wang et al., 2013). Lists detailing the advantages and disadvantages of materials and methods, operating principles, accuracy, and resolution of sensing have been described in reviews (Brites et al., 2012; Wang et al., 2013; Suzuki et al., 2016; Arai and Suzuki, 2018), as well as debates on single-cell thermometry (Baffou et al., 2014, 2015; Kiyonaka et al., 2015; Suzuki et al., 2015). Instead of repeating these details, this section introduces fluorescent nanoparticles that have been applied for probing purposes, including temperature sensing, with single particle analyses in physiological situations. Their functional ranges cover the temperature from ~25°C to ~42°C. Although the accuracy of determining the temperature depends on the optical setup that reads out signals from the probe, 0.5°C or better values are frequently reported. The nanoparticles can be further “functionalized” to target biological molecules and organelles.

### Fluorescent Polymeric Nanoparticles for Probing Temperature Changes

We have developed fluorescent polymeric nanoparticles that are able to probe temperature changes in single cells (Oyama et al., 2012; Takei et al., 2014). Each nanoparticle contains temperature-sensitive fluorophores such as Eu-thenolytrifluoroacetonate (EuTTA) or Eu-tris(dinaphthoylmethane)-bis-trioctylphosphine oxide) (EuDT), of which emission intensities inversely correlate with temperature changes. In some cases, less temperature-sensitive fluorophores were also embedded in the same polymeric matrix to form a nanoparticle, whose surface is further covered by a hydrophilic polymer outer layer (Takei et al., 2014; Arai et al., 2015; Ferdinandus et al., 2016). Measurement of the temperature can be achieved by determining the reduction of emission intensity of EuTTA or EuDT, or by the ratio of the intensity of EuTTA or EuDT to that of the less temperature-sensitive fluorophores which act as internal references. The nanoparticle thermosensor assures strong emission intensity since it can pack multiple fluorophores into one particle. Furthermore, it is capable of measuring temperature without being affected by various intracellular factors, such as pH, viscosity, ionic strength, etc.

### Quantum Dots

Qdots are fluorescent semiconductor nanocrystals which possess distinct optical and electrical properties. These properties render Qdots as a promising alternative to organic dyes in biological applications from cell biology to *in vitro* diagnostics. Qdots have broad excitation spectra and narrow emission spectra, which are composition- and size-dependent. Furthermore, the strong photoluminescence and high photostability of Qdots enable them to play an important role in single particle tracking (Michalet et al., 2005; Ichimura et al., 2014b; Pisanic et al., 2014). Applications in intracellular temperature measurement have also been reported using the shift of emission spectra caused by temperature changes (Yang et al., 2011; Tanimoto et al., 2016).

Functionalization is essential for Qdots to be applied to biological investigations. Being mostly synthesized in organic solvents, Qdots are too hydrophobic to be dissolved in aqueous buffers. Ligand exchange with hydrophilic compounds or encapsulation within amphiphilic coatings are therefore necessary to achieve solubility in water. Surface modification of Qdots provides an interface for conjugating to target molecules (cf. section Targeting).

Blinking and cytotoxicity are two major weak points of Qdots, which limit their biological and therapeutic applications. Blinking, also called intermittent fluorescence, is the phenomenon of a single Qdot particle exhibiting light and dark periods under continuous laser illumination (Ko et al., 2011). Blinking interferes with the tracing process in single-particle tracking studies. Both cadminium-containing and cadminium-free Qdots were found to induce the elevation of intracellular reactive oxygen species levels and increase cell apoptosis, which contributes to a decrease in cell viability (Derfus et al., 2004). Qdots in high concentrations also affect embryo development (Dubertret et al., 2002).

### Fluorescent Nanodiamonds

One of the newly emerging fluorescent probes is nanodiamonds containing nitrogen vacancy centers (NVCs) (Chang et al., 2018). An NVC is composed of a substitutional nitrogen adjacent to a defect in the lattice. The NVC excited by green light emits light in the range of red. Thus, the nanodiamonds containing NVCs are called fluorescent nanodiamonds. When NVCs are embedded within the nanodiamond they show an outstanding robustness to changes in environmental parameters (Sekiguchi et al., 2018). Similar to Qdots, fluorescent nanodiamonds are extremely photostable. In contrast to Qdots, no blinking is observed. Modification of the surface is also relatively easy (Sotoma et al., 2018); e.g., Tsai et al. successfully used this advantage to attach gold nanorods as tiny heat sources onto the surface of nanodiamonds to probe the local temperature at the heated dot (Tsai et al., 2017). Biocompatibility has been shown to be good. Bearing these advantages in mind, fluorescent nanodiamonds can be considered close to the ideal fluorescent probe.

There remain at least two issues regarding the use of fluorescent nanodiamonds as probes. Firstly, the NVC can be exposed at the surface of the nanodiamond, thus eradicating its robustness to the surrounding environment. Greater care should therefore be taken as the diameter of the nanodiamond gets smaller. Secondly, measurement can require long periods of time when extreme accuracy is required or when the amount of NVCs in the nanodiamond is reduced. For example, it is possible to measure the temperature using the negatively charged NVC, NV^−^. All optical methods detect changes of the emission peak by changing temperature (Plakhotnik et al., 2014). The temperature-dependent status of NV^−^ can also be detected by the downward peaks in emission intensity when the microwave is swept, known as optically detected magnetic resonance, or ODMR (Kucsko et al., 2013). Collecting enough photons or sweeping the microwave is required to draw spectra to determine the peaks. Efforts to shorten the sweeping process continue (Tzeng et al., 2015).

### Targeting

It is a challenging task to target nanoparticles to specific cells, organelles, or molecules within a living cell. Yet several attempts have been successful in biological uses. In *in vivo* conditions, a probe was prepared based on Qdots by immobilizing a ligand with a high affinity for hexahistidine (Arai et al., 2012). By using this Qdot probe, single molecule-tracking of his-tagged myosin V was achieved. Fujita et al. discovered the major sequestration mechanism for glucose transporter 4 in fully differentiated single 3T3L1 adipocytes (Fujita et al., 2010). Gonda et al. conjugated anti-protease-activated receptor1, a tumor cell membrane protein, with Qdots to uncover changes in membrane fluidity and morphology during tumor metastasis in living mice (Gonda et al., 2010). The extreme photostability of Qdots was one of the major reasons that these studies were made possible. More recently, single nanodiamonds surface functionalized with poly-dopamine followed by thiol terminated poly (ethylene glycol) were successfully targeted to single DNAs using the avidin-biotin system to track their locations (Jung et al., 2018).

There are other types of nanoparticles that are constructed step-by-step directly within living cells at the desired target regions. The first step is to prepare cells expressing single chain avidin (ScAVD) at the target region. The second and the third are to incubate the cells with dibenzocyclooctyne-biotin followed by azide-functionalized organic dyes to perform a copper-free click reaction to construct a ScAVD-biotin-functional dye conjugated nanoprobe (Hou et al., 2016a,b). These nanoprobes have increased brightness and photostability as well as improved localization specificity when compared to single fluorescent dyes.

## Imaging Technologies Without Labeling

Although labeling is a powerful technique to visualize the structure of the cell, it is not always preferred. Even when using a bio-safe dye, the detection of cancerous tissue using a labeling method during a surgical operation can significantly prolong the surgery, which increases the chances of complications. Labeling is also not preferred when producing cells for regenerative medicine. In this section, we introduce imaging techniques that don't require labeling. Raman microscopy and fluorescent lifetime imaging microscopy (FLIM) using auto-florescence are discussed (Figure 2).

### Raman Imaging Spectroscopy

When light enters a molecule, three types of scatters are generated, Reyleigh scattering, Stokes Raman scattering, and anti-Stokes Raman scattering. Reyleigh scattering has the same energy as incident light, whereas Stokes and anti-Stokes Raman scattering have less and more energy than incident light, respectively. The shift in wavelength (Raman shift) relates to the chemical bonds within the molecule, thus the molecule can be identified using the Raman spectrum. Raman spectroscopy is a non-labeling and non-destructive analysis method, which can obtain information relating to the chemical bonds of a molecule.

Raman imaging spectroscopy usually uses visible light for illumination so the optical resolution is generally higher than Infrared (IR) imaging spectroscopy. Other than standard Spontaneous Raman spectroscopy, there are several other modes of Raman imaging techniques, including Coherent anti-Stokes Raman spectroscopy (CARS), Surface-enhanced Raman spectroscopy (SERS), and Stimulated Raman spectroscopy (SRS). The various types of Raman spectroscopy are reviewed in Krafft and Popp (Krafft and Popp, 2015).

Raman spectra can be used to identify the state of the cell, thus, clinical diagnosis is possible at a tissue level. Raman spectroscopy has been used diagnostically for cancer (Austin et al., 2016; Santos et al., 2017), atherosclerosis (MacRitchie et al., 2018) and liver steatosis (Pacia et al., 2018). Raman imaging of living cells was difficult because the Raman signal is far weaker than fluorescence; strong illumination with a visible laser and a long exposure is therefore needed, which significantly damages the cell. However, recent advances in detectors, spectroscopes, and optical filters have enabled Raman imaging of living cells (Hamada et al., 2008). Now, cell state can be monitored using Raman spectroscopy at a single cell level (Ichimura et al., 2014a, 2015, 2016).

### Fluorescent Lifetime Imaging (FLIM)

FLIM is one of the most important technologies in optical microscopy, especially in live cell imaging, as fluorescence lifetime is independent of fluorophore concentration and focus drift. FLIM has been frequently used in Förester resonance energy transfer (FRET) based on fluorescent sensors that comprise two fluorescent proteins as a donor and an acceptor. The lifetime of the fluorescent protein is altered during energy transfer, thus a FRET sensor can be applied for FLIM. As such, small chemical sensors are also possible for FLIM, which are sensitive to the surrounding environment in the fluorescence lifetime domain. For successful examples, intracellular conditions such as levels of Ca^2+^ (Zheng et al., 2018), K^+^ (Paredes et al., 2013), Na^+^ (Roder and Hille, 2014), and Cl^−^ (Chao et al., 1989), as well as pH (Schmitt et al., 2014), and temperature (Itoh et al., 2016) can be monitored using FLIM. FLIM can also be performed in non-label imaging as there are naturally occurring endogenous fluorescent biomolecules, such as reduced nicotinamide adenine dinucleotide (phosphate) [NAD(P)H] and flavines present in the cell. Using FLIM, NADH, and NADPH can be distinguished and the relative ratio can be calculated, which is not possible using fluorescence intensity as their spectra are identical (Blacker et al., 2014).

FLIM using auto fluorescence, which is non-labeling, is especially useful in diagnostic purposes. Detection of macrophages in an atherosclerotic model (Marcu et al., 2005), glioma in brain tissue (Yong et al., 2006), retinal pigment epithelium (Miura, 2018), and basal cell carcinoma in skin (Galletly et al., 2008) are made possible using FLIM. Two-photon FLIM is preferred for tissue diagnostics purposes as it reduces photo-damage to the cells and reaches deeper within the tissue. Two-photon excitation is now used to distinguish between papillary and reticular dermis (Shirshin et al., 2017), in the diagnostics of inflamed human skin (Huck et al., 2016), and in melanoma detection (Seidenari et al., 2013). Second Harmonic Generation (SHG) and two-photon FLIM can be combined to increase the information obtained and used in diagnosis as the same laser source can be used (Provenzano et al., 2009; Ranjit et al., 2016).

### Other Non-label Imaging Technologies

There are other modes of non-label imaging technologies such as SHG and infrared (IR) spectroscopy. The information obtained by IR spectroscopy complements that obtained by Raman spectroscopy as when the IR signal from a particular chemical bond is strong, the signal obtained from Raman spectroscopy is weak, and vice versa. A number of products capable of obtaining Raman, FLIM, or IR are released every year, which will further advance this field.

## Summary

Nanotechnology provides tools to control, create, or cure biospecimens. The effect should be evaluated in a quantitative manner to gauge the efficacy of the methods or the quality of the results. Optical microscopy can provide essential information on how and why samples have reacted at sub-cellular resolution. Observation of morphological changes of cells (e.g., axon-like elongation for neuronal cells, multi-nucleus, and contractile structures in muscular cells, or lipid droplets in adipocytes) is one of the first steps to be performed. Probing intracellular reactions provides additional and much more detailed insights. In this article, we reviewed optical methods to probe intracellular events at sub-cellular resolutions in living cells using optical microscopes. Some methods use fluorescent nanoprobes, several of which are designed to change their properties in fluorescence in response to changes in the chemical and physical conditions surrounding the probe. Other methods do not require these tiny probes, but are performed in purely optical manner. Considering the ease of availability, optical microscopy should continue to be a standard method. With the wide variety in probes as well as in equipment, methods are available to meet a range of specific needs. Optimal choices will always be dependent on the sample being studied.

## Author Contributions

HF, SA, and MS conceived the story. All the authors co-wrote and critically revised the manuscript.

### Conflict of Interest Statement

The authors declare that the research was conducted in the absence of any commercial or financial relationships that could be construed as a potential conflict of interest.
